# CoQ10 Deficiency May Indicate Mitochondrial Dysfunction in Cr(VI) Toxicity

**DOI:** 10.3390/ijms18040816

**Published:** 2017-04-24

**Authors:** Xiali Zhong, Xing Yi, Rita de Cássia da Silveira e Sá, Yujing Zhang, Kaihua Liu, Fang Xiao, Caigao Zhong

**Affiliations:** 1 Department of Health Toxicology, School of Public Health, Central South University, Changsha 410008, China; xializhong87@sina.com (X.Z.); yistar@163.com (X.Y.); zhangyujing24@163.com (Y.Z.); lecapher@gmail.com (K.L.); fangxiao@csu.edu.cn (F.X.); 2 Department of Environmental Health Science, Bloomberg School of Public Health, Johns Hopkins University, Baltimore, MD 21205, USA; 3 Department of Physiology and Pathology, Health Sciences Center, Federal University of Paraíba, 58059-900 João Pessoa, Brazil; ritacassia.sa@bol.com.br

**Keywords:** hexavalent chromium Cr(VI), coenzyme Q10, reactive oxygen species (ROS), mitochondrial membrane potential (MMP), L-02 hepatocytes, apoptosis

## Abstract

To investigate the toxic mechanism of hexavalent chromium Cr(VI) and search for an antidote for Cr(VI)-induced cytotoxicity, a study of mitochondrial dysfunction induced by Cr(VI) and cell survival by recovering mitochondrial function was performed. In the present study, we found that the gene expression of electron transfer flavoprotein dehydrogenase (ETFDH) was strongly downregulated by Cr(VI) exposure. The levels of coenzyme 10 (CoQ10) and mitochondrial biogenesis presented by mitochondrial mass and mitochondrial DNA copy number were also significantly reduced after Cr(VI) exposure. The subsequent, Cr(VI)-induced mitochondrial damage and apoptosis were characterized by reactive oxygen species (ROS) accumulation, caspase-3 and caspase-9 activation, decreased superoxide dismutase (SOD) and ATP production, increased methane dicarboxylic aldehyde (MDA) content, mitochondrial membrane depolarization and mitochondrial permeability transition pore (MPTP) opening, increased Ca^2+^ levels, Cyt c release, decreased Bcl-2 expression, and significantly elevated Bax expression. The Cr(VI)-induced deleterious changes were attenuated by pretreatment with CoQ10 in L-02 hepatocytes. These data suggest that Cr(VI) induces CoQ10 deficiency in L-02 hepatocytes, indicating that this deficiency may be a biomarker of mitochondrial dysfunction in Cr(VI) poisoning and that exogenous administration of CoQ10 may restore mitochondrial function and protect the liver from Cr(VI) exposure.

## 1. Introduction

Chromium (Cr) and its compounds have become a serious public health issue, causing environmental pollution [1,2] and threatening human health. The health hazards associated with exposure to Cr are dependent on its oxidation state [3,4], with hexavalent chromium Cr(VI) being the most toxic component. Throughout the world, human exposure occurs mainly via industrial uses, such as leather tanning and steel manufacturing, as well as in food additives and tobacco [5,6]. Another source of contact is drinking water contaminated with Cr(VI). In vivo and in vitro studies have demonstrated that Cr(VI) can cause a wide range of toxic effects, including hepatotoxicity, in animals and humans [1,2,3,4]. It has been reported that the liver shows the highest accumulation following oral exposure to Cr(VI) [7,8,9]. The liver is the primary organ involved in xenobiotic metabolism and, for this reason, is particularly susceptible to injury. However, the liver as a target organ for Cr(VI) after oral exposure in humans remains controversial. For instance, Proctor et al. reported that Cr(VI) is not carcinogenic to humans via the oral route of exposure at permissible drinking water concentrations [10]. Several other studies have suggested that Cr(VI) could induce liver injury [1,3,11] and may cause primary cancer or increase the risk of liver cancer [12,13,14]. The increasing incidence of Cr(VI)-induced hepatotoxicity has emphasized the importance of elucidating the intoxication mechanism and identifying useful antidotes for Cr(VI) toxic effects on the liver.

Cr(VI) can easily enter cells through anion channels, and, once inside, it is reduced to its intermediate metabolites, Cr(IV), Cr(V), and the more stable form Cr(III), by enzymatic and non-enzymatic reductants [15]. Reactive oxygen species (ROS) are generated in the oxidation–reduction process and play a critical role in the mechanism of Cr(VI)-induced cytotoxicity. ROS accumulation, for instance, is known to cause the collapse of mitochondrial membrane potential and the opening of the mitochondrial permeability transition pore (MPTP) [16]. Cr(VI) induces cell apoptosis through intrinsic and extrinsic pathways involving the release of cytochrome c (Cyt c) from the mitochondrial intermembrane space. The release of Cyt c from the mitochondrial intermembrane space is regulated by B-cell lymphoma-2 (Bcl-2) family proteins, including anti-apoptotic (such as Bcl-2 and Bcl-xl) and pro-apoptotic proteins (such as Bcl-xs, Bax, and Bid) [17]. It has been postulated that the ratio of anti- and pro-apoptotic Bcl proteins regulates the function of MPTP within the mitochondria. Cr(VI)-induced ROS accumulation might cause an imbalance in Bcl-2 family proteins, swelling of the mitochondrial membrane, opening of the MPTP, release of Cyt c into the cytoplasm, and activation of caspase-9 and -3 [17,18], which would eventually trigger cell apoptosis.

Coenzyme Q (CoQ10) is an essential endogenous molecule in cell respiration and metabolism. It functions as a mitochondrial antioxidant, inhibiting lipid peroxidation and scavenging free radicals, as well as maintaining genome stability [19]. Moreover, CoQ10 possesses an independent anti-apoptosis function that regulates MPTP [20]. CoQ10 biosynthesis occurs in the mitochondrial matrix through the mevalonate pathway [21]. Evidence suggests that mutations in the genes involved in the biosynthesis of CoQ10 could cause primary and secondary CoQ10 deficiencies. They have also been linked to various clinical mitochondrial diseases [22,23]. CoQ10 deficiency could disturb mitochondrial bioenergetics and oxidative stress, as demonstrated by decreased ATP generation, increased ROS production, and cell death [24,25]. In general, secondary CoQ10 deficiency may be induced by dietary insufficiency or exposure to certain xenobiotics [26,27]. Several studies have shown that CoQ10 is susceptible to environmental toxins, which cause CoQ10 deficiency at both the cellular and in vivo levels [28,29]. Considering the pivotal role played by CoQ10 in mitochondrial function, this study aimed to investigate whether Cr(VI) can induce changes in the level of CoQ10, and whether CoQ10 treatment is effective against Cr(VI)-induced hepatotoxicity.

## 2. Results

### 2.1. Effect of Coenzyme Q10 and Cr(VI) on L-02 Hepatocyte Viability

**Figure 1:**
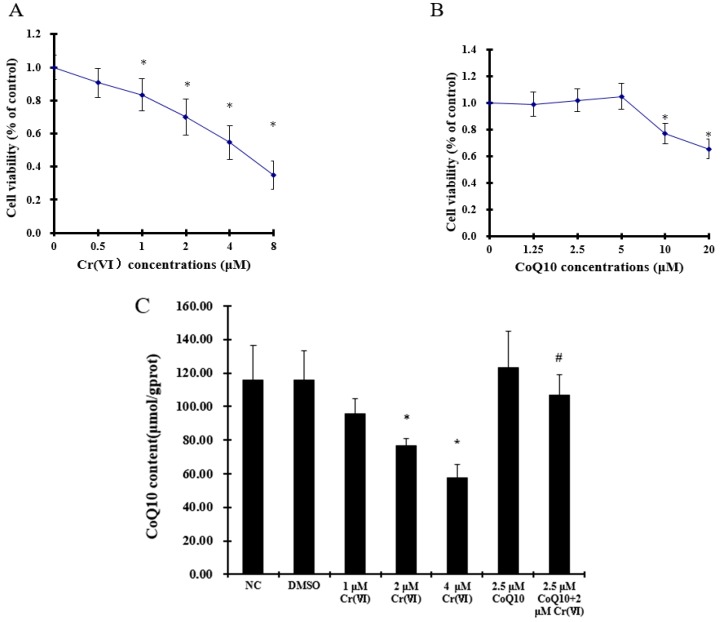
(**A**) Effect of different doses of Cr(VI) exposure on L-02 hepatocyte viability. Cells were cultured with different concentrations of Cr(VI), and the cell viability was determined by the 3-(4,5-dimethylthiazol-2-yl)-2,5-diphenyltetrazolium bromide (MTT) assay as described previously; (**B**) Effect of different doses of CoQ10 exposure on L-02 hepatocyte viability; (**C**) The CoQ10 content in the mitochondria of L-02 hepatocytes treated with CoQ10 and Cr(VI). The data were presented as mean ± SD (*n* = 6). * *p* < 0.05 compared with the control group; # *p* < 0.05 compared with the 2 μM Cr(VI) treatment group.

To assay the changes in cell viability after exposure to Cr(VI), we evaluated the dose effects of Cr(VI) on cultured L-02 hepatocytes with or without CoQ10 pretreatment. It was observed that increasing Cr(VI) concentrations of 0.5, 1, 2, 4, and 8 μM significantly decreased cell viability (*p* < 0.05), as shown in Figure 1A. Treatment with CoQ10 at 0–5 μM increased cell viability, although not at a statistically significant level (*p* > 0.05). In contrast, treatment with concentrations of 5–20 μM CoQ10 significantly decreased cell viability (*p* < 0.05), as shown in Figure 1B.

### 2.2. Cr(VI) Decreases CoQ10 Content in L-02 Hepatocytes

The assays of CoQ10 level showed that Cr(VI) decreased the CoQ10 concentration in the mitochondria of L-02 hepatocytes when compared with the control group (*p* < 0.05). Pretreatment with CoQ10 restored the level of CoQ10 compared with the 2 μM Cr(VI) treatment group (*p* < 0.05), as shown in Figure 1C.

### 2.3. Effect of Cr(VI) on the Expression of Genes Involved in the CoQ10 Synthesis Pathway

Because Cr(VI) treatment decreased the level of CoQ10, and to understand the mechanism of CoQ10 deficiency, we analyzed the expression of genes involved directly or indirectly in the CoQ10 synthesis pathway. As shown in Table 1, electron transfer flavoprotein dehydrogenase (*ETFDH*) was the gene most strongly downregulated by Cr(VI) (log2(Ratio) = −1.41). *PDSS2*, *COQ5*, and *COQ9* were also downregulated by Cr(VI), but the fold changes were less than 2 (log2(Ratio) = −0.92, −0.99, and −0.94, respectively). Interestingly, aarF domain-containing kinase 3 (*ADCK3*) was upregulated after exposure to Cr(VI) (log2(Ratio) = 0.97). No significant changes were observed in the other genes. These results indicated that Cr(VI) affected the expression of genes directly or indirectly involved in the CoQ10 synthesis pathway to cause CoQ10 deficiency.

### 2.4. Oxidative Damage Induced by Cr(VI) Is Reduced by CoQ10

**Figure 2:**
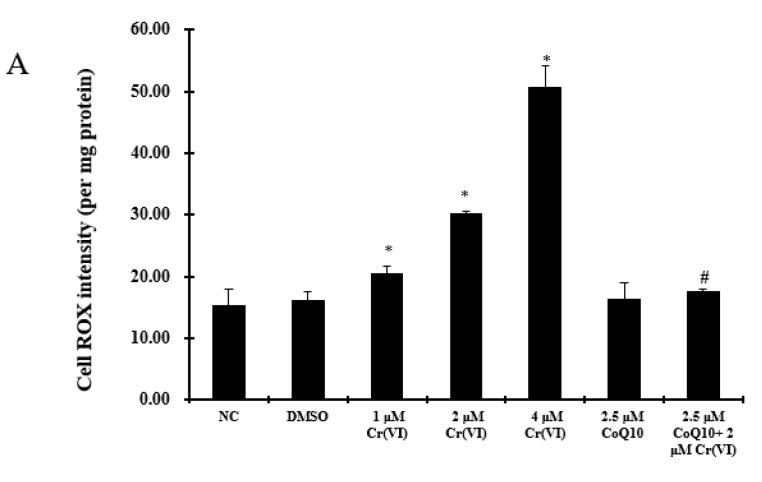

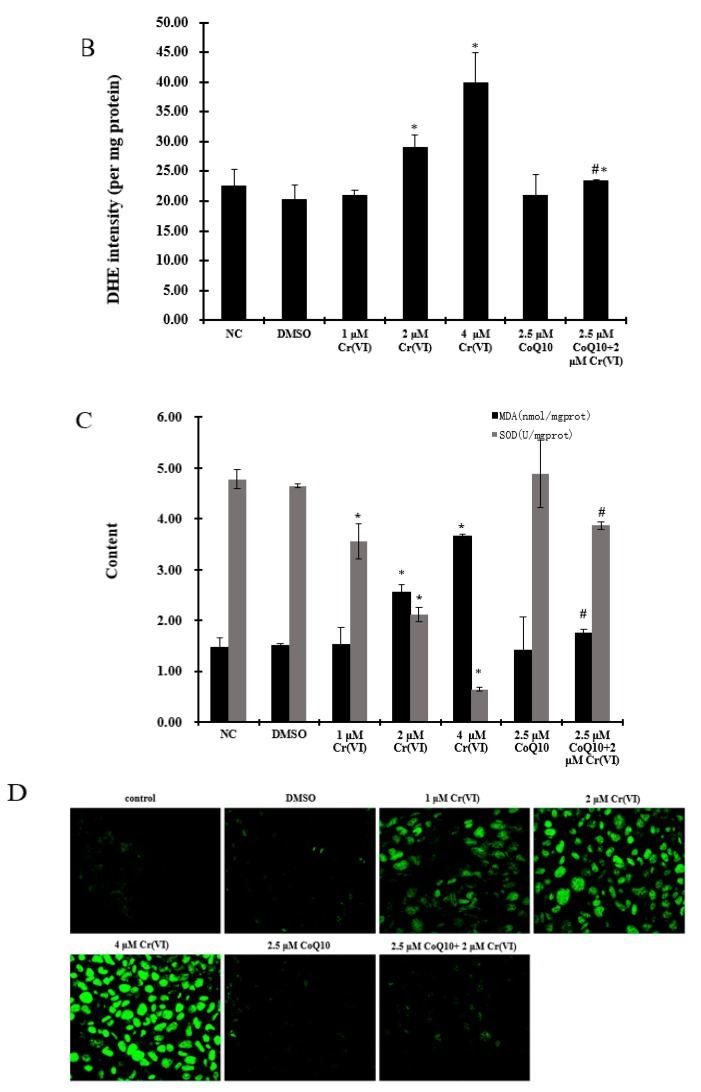
CoQ10 attenuates oxidative damage induced by Cr(VI). (**A**) Quantification of ROS levels. Effect of CoQ10 on Cr(VI)-induced ROS accumulation in L-02 hepatocytes and quantitation by fluorescence spectrophotometry; (**B**) After L-02 hepatocytes were treated with Cr(VI) (0~4 μM) for 24 h, with or without CoQ10 pretreatment for 2 h, O_2_^−^ generation was detected with dihydroethidium; (**C**) CoQ10 reduced the oxidative damage induced by Cr(VI). The cells were treated with Cr(VI) (0~4 μM) for 24 h, with or without CoQ10 pretreatment, and MDA was detected by the MDA detection kit as the end product of lipid oxidation. SOD was measured using the total superoxide dismutase activity assay, which involves the inhibition of superoxide-induced chromogen chemiluminescence by SOD; (**D**) Effect of CoQ10 on Cr(VI)-induced ROS accumulation. The cells were incubated with 5 μM of CellROX^®^ Green Reagent for 30 min and observed under a confocal microscope using a 40× objective. Brighter green fluorescence indicated greater ROS accumulation. The data are presented as mean ± SD (*n* = 6). * *p* < 0.05 compared with the control group; # *p* < 0.05 compared with the 2 μM Cr(VI) treatment group.

We measured ROS production using dihydroethidine (DHE) and CellROX^®^ Green Reagent, which detect superoxide and ROS, respectively. Cr(VI) significantly enhanced ROS and O_2_^−^ generation compared with the control group (*p* < 0.05). Pretreatment with 2.5 μM CoQ10 prevented Cr(VI)-induced ROS accumulation and excessive O_2_^−^, as shown in Figure 2A,B. The levels of ROS in the CoQ10-treated group did not significantly change in comparison with those of the normal control group (*p* > 0.05). SOD is an enzyme that plays an important role in the protection of the cell membrane against oxidative stress. It can catalyze the dismutation of O_2_^−^ to O_2_ and to the less reactive species H_2_O_2_. Treatment with Cr(VI) caused a significant decrease in SOD levels when compared to control values. No significant difference in SOD activity was observed between the Cr(VI) group pretreated with CoQ10 and the control group (*p* < 0.05), as shown in Figure 2C. In addition, CoQ10 effectively restored the SOD level to protect hepatocytes against oxidative damage induced by Cr(VI). MDA was evaluated as an indicator of hepatocyte lipid peroxidation. Cr(VI) significantly increased MDA levels compared with the control group (*p* < 0.05), and pretreatment with CoQ10 markedly reduced the level of Cr(VI)-induced MDA (*p* < 0.05) (Figure 2C).

### 2.5. Induction of Mitochondrial Loss by Cr(VI) Can Be Counteracted by Supplementation with CoQ10

**Figure 3:**
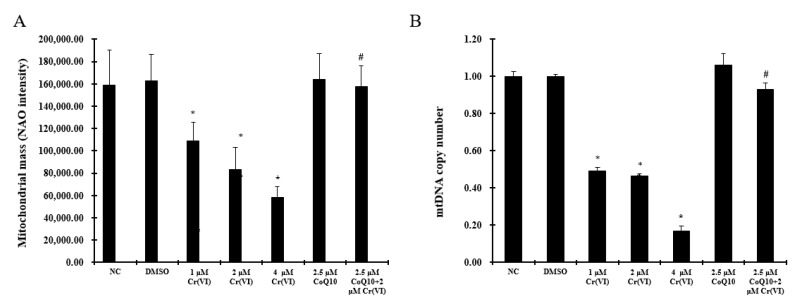
Cr(VI) triggers significant mitochondrial biogenesis loss. (**A**) NAO staining was used to analyze the mitochondrial mass using a microplate reader; (**B**) quantitative real-time PCR analysis was applied to detect the mtDNA copy number. The data are presented as mean ± SD (*n* = 6). * *p* < 0.05 compared with the control group; # *p* < 0.05 compared with the 2 μM Cr(VI) treatment group.

Mitochondrial loss was reflected by a decrease in the mitochondrial mass and mtDNA. We examined the mitochondrial mass using the 10-*N*-nonyl acridine orange (NAO) fluorescence intensity, which was lower in the different Cr(VI) concentration groups than in the control group. CoQ10 maintained the mitochondrial mass at a normal level against Cr(VI) exposure (Figure 3A). In addition, Cr(VI) treatment markedly reduced the mtDNA copy number, and CoQ10 preserved the mtDNA copy number (Figure 3B).

### 2.6. Cr(VI) Induces Mitochondrial Depolarization, MPTP Opening, Ca^2+^ Overload, and Decreased ATP Levels, and These Outcomes Are Attenuated by CoQ10

**Figure 4:**
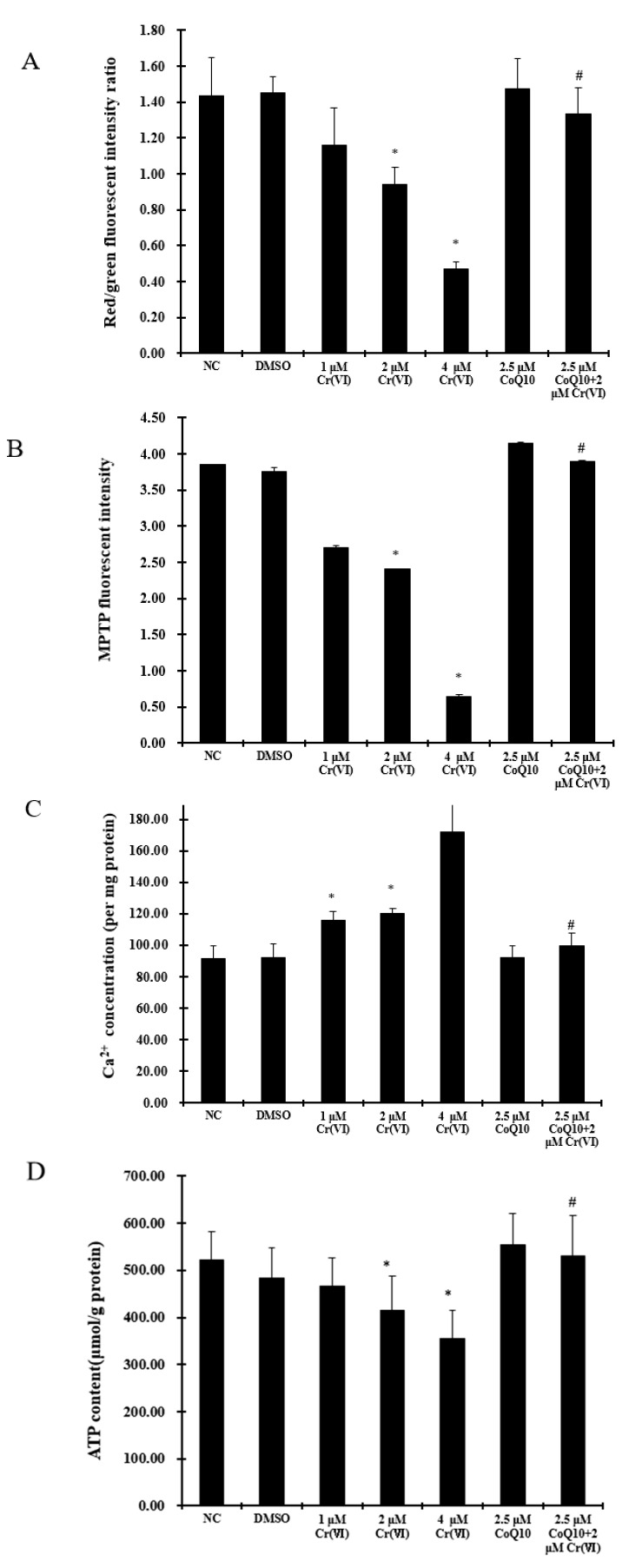
Cr(VI) induces mitochondrial depolarization, MPTP opening, Ca^2+^ overload, and ATP level decrease, and these outcomes are attenuated by CoQ10. (**A**) Effect of CoQ10 on Cr(VI)-increased mitochondrial membrane potential in L-02 hepatocytes. The mitochondrial membrane potential was examined by JC-1 staining; (**B**) The activity of MPTP was detected using the calcein-AM-cobalt assay; (**C**) the Ca^2+^ concentration was measured with Flo-3M by fluorescence spectrophotometry; (**D**) cells were treated with Cr(VI) (0–4 μM) for 24 h, with or without CoQ10 pretreatment for 2 h, and the ATP levels in L-02 hepatocytes were assessed. The data are presented as mean ± SD (*n* = 6). * *p* < 0.05 compared with the control group; # *p* < 0.05 compared with the 2 μM Cr(VI) treatment group.

The effect of Cr(VI) on the mitochondrial membrane potential (MMP) was quantified by the uptake of JC-1, as illustrated in Figure 4A. The shift in the membrane potential was observed as the disappearance of fluorescent red/green-stained mitochondria, showing a large negative MMP, and as the increase in fluorescent green-stained mitochondria, indicating the loss of MMP. With increasing Cr(VI) concentrations, MMP significantly decreased as compared to the normal controls (*p* < 0.05), indicating mitochondrial membrane depolarization. Pretreatment with CoQ10 relieved Cr(VI)-induced mitochondrial membrane depolarization. To directly assess MPTP opening, the calcein-AM-cobalt assay was performed in L-02 hepatocytes. The inner mitochondrial membrane permeability was significantly increased in response to Cr(VI) stimuli in a concentration-dependent manner. The degree of MPTP opening in cells after co-incubation with 2 μM Cr(VI) and 2.5 μM CoQ10 was significantly decreased compared with cells incubated with 2 μM Cr(VI) alone (Figure 4B). MPTP opening is a Ca^2+^-dependent event; hence, to assess the Ca^2+^ concentration in L-02 hepatocytes, as shown in Figure 4C, the cells were incubated with the Flo-3M probe to detect the intercellular Ca^2+^ concentration. Cr(VI) caused a statistically significant Ca^2+^ concentration-dependent increase (*p* < 0.05), while pretreatment with CoQ10 significantly attenuated Ca^2+^ overload when compared with treatment with 2 μM Cr(VI) alone (*p* < 0.05). These results suggest that CoQ10 suppressed Ca^2+^ overload and maintained a suitable degree of MPTP opening. Apoptosis induced by toxicants is an energy-consuming process and thus is accompanied by a massive decrease in cellular ATP production. To examine the effects of Cr(VI) on mitochondrial ATP production, L-02 hepatocytes were treated with 1, 2, or 4 μM Cr(VI) for 24 h or pretreated with 2.5 μM CoQ10 for 30 min, followed by the addition of 2 μM Cr(VI). ATP levels were then measured colorimetrically. As shown in Figure 4D, Cr(VI) induced a statistically significant and concentration-dependent decrease in ATP in L-02 hepatocytes (*p* < 0.05). Pretreatment with CoQ10 significantly decreased the Cr(VI)-induced decrease in ATP (*p* < 0.05).

### 2.7. Cr(VI) Induces Cyt c Release, Caspase-3 and Caspase-9 Activation, and Unbalanced Bcl-2/Bax Expression in Response to Apoptotic Stimuli, and CoQ10 Counteracts These Outcomes

**Figure 5:**
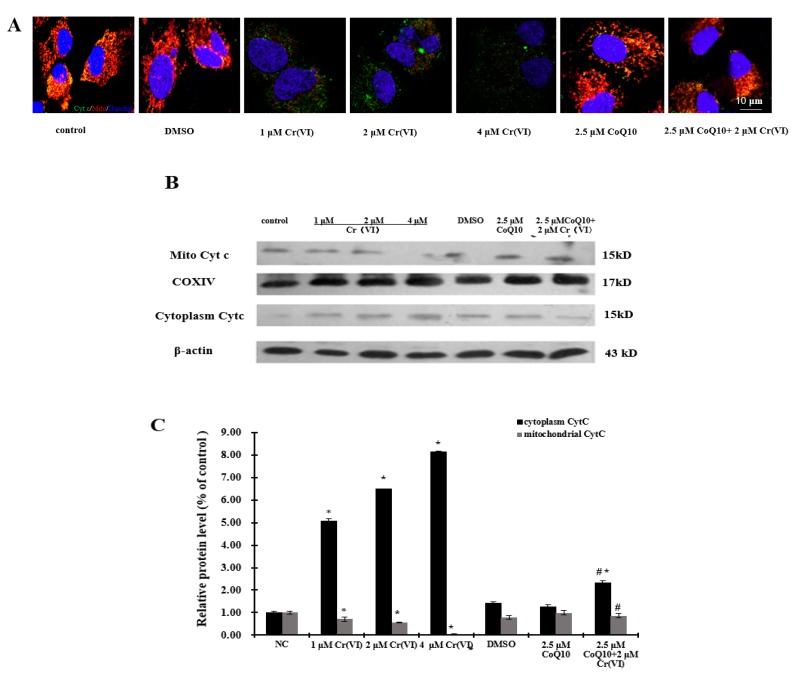
Cr(VI) induces cytochrome c release from the mitochondria to the cytoplasm. (**A**) Merged images of the mitochondria (red), Cyt c (green) and nucleus (blue) after exposure to Cr(VI) for 24 h in L-02 hepatocytes. Cyt c (green) and mitochondria (red) localization (yellow) indicates that Cyt c is still inside mitochondria. The separation of Cyt c and mitochondria suggests that Cyt c is no longer within the mitochondria and has been released into the cytoplasm, scale bar: 10 μm; (**B**) CoQ10 prevents Cyt c release to the cytoplasm; Cyt c protein expression was measured by Western blotting. COXIV and β-actin were used as loading controls; (**C**) The relative protein levels were calculated by Image J software. Experiments were repeated three times and showed similar results.

**Figure 6:**
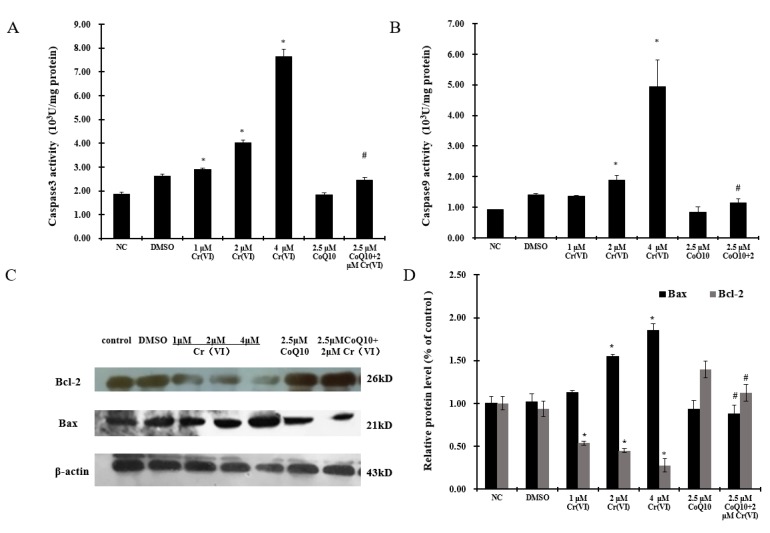
Cr(VI) induces caspase-3 and caspase-9 activation and unbalanced Bcl-2/Bax expression in response to apoptotic stimuli, and CoQ10 counteracts these outcomes. Cells were treated with Cr(VI) (0~4 μM) for 24 h, with or without CoQ10 pretreatment for 2 h. Caspase-3 (**A**) and -9 (**B**) activities were detected using a microplate reader; (**C**) The expression of Bcl-2 and Bax was measured by Western blotting and the relative protein levels were calculated by Image J software (**D**). The data are expressed as mean ± SD (*n* = 6). * *p* < 0.05 compared with the control group; # *p* < 0.05 compared with the 2 μM Cr(VI) treatment group.

Cyt c, caspase-3 and caspase-9 activities were analyzed as indexes of apoptosis execution via the intrinsic (mitochondrion-dependent) pathway. Figure 5 shows the release of Cyt c into the cytoplasm. At 24 h after application of Cr(VI), cytoplasmic Cyt c levels were markedly increased but remained substantially unaffected if treatment was preceded by CoQ10 administration. Similarly, Figure 6A,B show that caspase-3 and caspase-9 activities were enhanced at 24 h after Cr(VI) exposure. The enhancement was dramatically lower when Cr(VI) exposure was preceded by CoQ10 administration. CoQ10 had the ability to prevent Cyt c release, and caspase-3 and caspase-9 activation in response to Cr(VI) exposure, three events that are triggered by MPTP opening. CoQ10 inhibited apoptosis by directly maintaining MPTP in the closed conformation. Additionally, the process of apoptosis is regulated by the Bcl-2 family of proteins, which includes anti-apoptotic and pro-apoptotic proteins. As shown in Figure 6C,D, 24-h exposure to different concentrations of Cr(VI) induced significant concentration-dependent inhibition of Bcl-2 and induction of Bax. Pretreatment with 2.5 μM CoQ10 restored Bcl-2 expression and decreased Bax expression compared with treatment with 2 μM Cr(VI) alone.

### 2.8. Cr(VI) Induces L-02 Hepatocyte Apoptosis in a Concentration-Dependent Manner, and CoQ10 Might Reduce the Rate of Apoptosis

**Figure 7:**
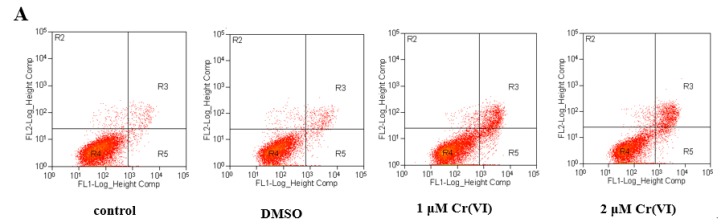

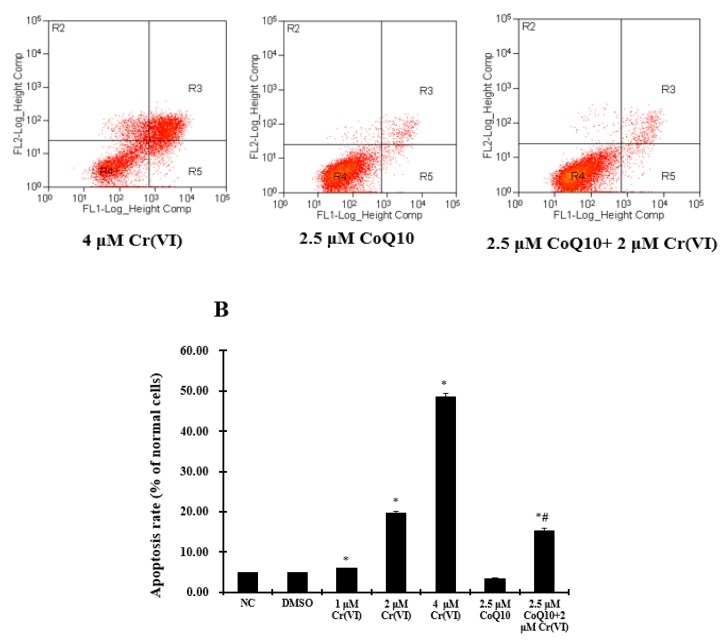
Cr(VI) induces apoptosis in L-02 hepatocytes, and CoQ10 antagonizes apoptosis. (**A**) Cells were stained with Annexin V-FITC/PI and analyzed by flow cytometry. Both early apoptotic and late apoptotic cells were assessed in the cell death determinations. The experiments were repeated three times; (**B**) Quantification of apoptotic cells. Data were obtained from flow cytometry assays and were expressed as mean ± SD (*n* = 6). * *p* < 0.05 compared with the control group; # *p* < 0.05 compared with the 2 μM Cr(VI) treatment group.

To measure the effects of CoQ10 on programmed cell death after Cr(VI) exposure, we analyzed cell apoptosis using the Annexin V-FITC and propidium iodide (PI) double staining methods after incubation for 30 min. As shown in Figure 7A,B, 24 h exposure of Cr(VI) increased the early and late apoptotic populations in L-02 hepatocytes. Approximately 5.95%–48.46% of the cell population expressed high FITC and low PI signals, which are indicative of apoptotic cells, following treatment with up to 4 μM Cr(VI). Pretreatment with CoQ10 attenuated the Cr(VI)-induced increase in Annexin V-positively stained cells. The protective effect of CoQ10 on Cr(VI)-induced apoptosis was 15.28% (*p* < 0.05), indicating that CoQ10 can attenuate Cr(VI)-induced apoptosis.

## 3. Discussion

In the present study, we demonstrated that Cr(VI)decreased the level of endogenous CoQ10 by disturbing the CoQ10 synthesis pathway, and that the pretreatment with CoQ10 maintained the level of endogenous CoQ10. These findings led us to investigate the role of CoQ10 in the mechanism of Cr(VI)-induced hepatotoxicity and its possible role as a hepatoprotective agent against Cr(VI)-induced hepatocyte damage.

In order to achieve these goals, we examined the genes involved both directly and indirectly in the CoQ10 biosynthetic pathway. Cr(VI) changed the expression of many genes, including *ETFDH*, which exhibited the strongest downregulation. *ETFDH* is indirectly involved in the biosynthesis of CoQ10 and encodes a component of the electron-transfer system in mitochondria. Gempel et al. reported that mutations in the *ETFDH* gene cause pure myopathy, as evidenced in seven patients from five different families with severely decreased activities of respiratory chain complexes I and II + III and CoQ10 deficiency [30]. We also previously demonstrated that Cr(VI) induces mitochondrial dysfunction by disturbing electron transport and inhibiting the respiratory chain complexes. As a link to our present study, we tentatively propose that Cr(VI) may cause secondary deficiency of CoQ10 in L-02 hepatocytes, resulting in mitochondrial dysfunction and cell apoptosis. This result provides a new perspective on the mechanism of Cr(VI)-induced hepatotoxicity.

In addition, Cr(VI) downregulated the expression of *PDSS2*, *COQ5*, and *COQ9*, which are directly involved in the CoQ10 biosynthesis pathway. Defects in these genes are a cause of CoQ10 deficiency [31]. *COQ5* catalyzes the only C-methylation step in the CoQ10 biosynthesis pathway in yeast. Chen et al. demonstrated that an uncoupling chemical in CoQ10 dose deficiency downregulates the mature form of *COQ5*. They also showed that knockdown of the *COQ5* gene reduces CoQ10 levels, indicating that *COQ5* plays a critical role in the biosynthesis of CoQ10 [32]. In the present study, Cr(VI) suppressed *COQ5* gene expression, but not strongly. It is possible that Cr(VI) induced *ETFDH* inhibition, causing mitochondrial dysfunction to disturb the expression of genes in CoQ10 biosynthesis. This is supported by Hsiu-Chuan’s study, which reported the suppressive effect of FCCP on *COQ5* levels in association with decreased mitochondrial membrane potential, mitochondrial ATP production, and CoQ10 levels [33]. Interestingly, *ADCK3* was upregulated after exposure to Cr(VI), although not significantly. *ADCK3* is required for the biosynthesis of CoQ10, and mutation of *ADCK3* has been associated with CoQ10 deficiency in humans [34]. *ADCK3* also functions in an electron-transferring membrane protein complex in the respiratory chain. Tumor suppressor p53 can induce *ADCK3* expression, and in response to DNA damage, inhibition of *ADCK3* expression partially suppresses p53-induced apoptosis [35]. We believe that CoQ10 maintains homeostasis only when gene expression is maintained at normal levels. However, little is currently known about the regulation of CoQ10 gene expression. Here, we present preliminary data that mitochondrial CoQ10 deficiency may represent a potential biomarker of Cr(VI) toxicity. However, to better understand the mechanism of Cr(VI)-induced CoQ10 deficiency, more robust evidence is needed.

Mitochondrial loss has been indicated to play a prominent role in mitochondrial dysfunction. Our results showed that Cr(VI) exposure led to mitochondrial loss in hepatocytes, as reflected by a decrease in the mitochondrial mass, mtDNA copy number, and inhibition of expression of components of the mitochondrial respiratory chain [36]. The observed Cr(VI)-induced decrease in the level of mtDNA copy number supports a link between CoQ10 deficiency and mtDNA depletion [37,38]. Moreover, mtDNA is vulnerable to ROS due to the lack of protection from histones and a self-repair mechanism. Our results also sowed Cr(VI)-induced CoQ10 deficiency and ROS accumulation. It is reported that CoQ10 deficiency may cause mitochondrial dysfunction, thus triggering ROS generation. The capability of ROS scavenging is weakened, possibly further aggravating ROS accumulation due to CoQ10 deficiency [39]. Supplementation with CoQ10 could increase the mitochondrial mass, mtDNA copy number, and mitochondrial electron transport chain activity, as demonstrated by our study and Duberley’s research [40]. Previous investigations have also shown that CoQ10 protects against neuron apoptosis induced by iron by reducing ROS accumulation and inhibiting lipid peroxidation [41]. Consistent with previous studies, we found that pretreatment with CoQ10 eliminated excessive ROS and O_2_^−^, reduced lipid peroxidation, and maintained SOD content. SOD plays an important role in the protection of cell membranes against oxidative stress by catalyzing the dismutation of O_2_^−^ to O_2_ and to the less reactive species H_2_O_2_ [42], corroborating the findings that CoQ10 plays a critical role in Cr(VI)-induced mitochondrial dysfunction.

Mitochondria are the factory of ATP production via oxidative phosphorylation [43]. In return, the primary function of mitochondria and homeostasis are maintained by adequate ATP levels. CoQ10 is an essential cofactor of oxidative phosphorylation to permit ATP biosynthesis [44]. Our data indicated that Cr(VI) causes CoQ10 deficiency and markedly reduces cellular ATP. ATP production is also related to Ca^2+^ and ROS generation. Calcium overload has been proposed to play a crucial role in ROS generation [45], which could aggravate mitochondrial damage in hepatocytes. Furthermore, Ca^2+^ induces Cyt c release from mitochondria by enhancing Cyt c dislocation, competing for cardiolipin-binding sites in the mitochondrial inner membrane, or activating MPTP opening, resulting in the disturbance of electron transfer and the Q cycle. As a consequence, there is an upsurge in ROS accumulation [46]. Therefore, eliminating Ca^2+^ overload and ROS accumulation are conducive to promoting ATP synthesis and repairing damaged mitochondria. In this study, we have demonstrated that CoQ10 could attenuate Cr(VI)-induced adverse effects by preventing Ca^2+^ overload and scavenging excessive ROS.

Apoptosis is an energy-consuming process that is regulated by the activation of caspases [47]. Cr(VI) induced the release of Cyt c from mitochondria, accompanied by activation of caspase-3 and 9, which are associated with significantly reduced Bcl-2 expression and enhanced Bax expression. Previous studies have confirmed that, after Cyt c is released from the mitochondrial intermembrane space into the cytosol, it forms a complex with Apaf-1 and pro-caspase, thereby triggering caspase-9 activation and the consequent initiation of the caspase cascade and induction of cell apoptosis [48]. In addition, cell apoptosis is regulated by the balance of pro- and anti- apoptotic proteins. It is generally believed that the anti-apoptotic protein Bcl-2 counterbalances oxidative damage and maintains the structural and functional integrity of the mitochondrial membrane by preventing Cyt c release. The Bax protein exerts an important effect in cell apoptosis. High Bax expression levels and the formation of homo- or heterodimers with Bcl-2 lead to cell death [49]. We also saw that pretreatment with CoQ10 could block the release of Cyt c, inhibit the activation of caspase-3 and caspase-9, and regulate the expression of Bcl-2/Bax to reach equilibrium and prevent subsequent apoptosis in L-02 hepatocytes. These results are compatible with the findings of other studies using CoQ10 against iron-induced neuronal toxicity [28], statin toxicity in hepatocytes [50], or ethanol-induced apoptosis in corneal fibroblasts [51].

As mentioned above, CoQ10 could alleviate Cr(VI)-induced hepatotoxicity. However, the mechanism of the protective effects of CoQ10 is not fully understood. Possible reasons for the protective effects of CoQ10 in Cr(VI)-induced toxicity include the fact that Cr(VI) is a water-soluble compound that can be easily reduced by water-soluble antioxidants [52]. Therefore, Cr(VI) might be reduced to Cr(III) before entering the cell when co-incubated with CoQ10. However, considering that Cr(VI) can dissolve in the medium, CoQ10, being a lipid-soluble quinone, may fail to interact with Cr(VI) outside the cell. On the other hand, since the cells were pretreated with CoQ10 for 2 h prior to exposure to Cr(VI), it is possible that CoQ10 may have entered the cell, and CoQ10 was distributed on membranes to block the opening of anion channels to prevent the entrance of Cr(VI) into the cell. This is supported by several studies showing that quinones modulate MPTP through a common binding site rather than through redox reactions [53,54]. This study also demonstrated that CoQ10 could decrease the MPTP opening degree to antagonize the mitochondrial toxicity induced by Cr(VI). Moreover, considering that exogenous CoQ10 enhanced the level of CoQ10 in mitochondria, we propose that the protective effect of CoQ10 might be associated with its role as a mobile electron transporter. CoQ10 can correct the disorder of electron transfer and improve the Q cycle, thus attenuating Ca^2+^ overload and Cyt c release. We have shown that CoQ10 can prevent cell apoptosis, but the results did not indicate how CoQ10 plays a protective role against Cr(VI)-induced hepatotoxicity. The detoxification role of CoQ10 against Cr(VI)-induced hepatotoxicity should be more comprehensively investigated.

## 4. Materials and Methods

### 4.1. Materials

Potassium dichromate (K_2_Cr_2_Q_7_), coenzyme Q10, 3-(4,5-dimethylthiazol-2-yl)-2,5-diphenyl-tetrazolium bromide (MTT), and dimethyl sulfoxide (DMSO) were purchased from Sigma (St. Louis, MO, USA). RPMI-1640 culture medium, fetal bovine serum (FBS), trypsin, and penicillin-streptomycin were provided by Dingguo Changsheng Biotechnology Co. LTD (Beijing, China). All solvents and chemicals were analytical grade.

### 4.2. Cell Culture

The normal liver L-02 cell line (Type Culture Collection of the Chinese Academy of Sciences, Shanghai, China) was derived from adult human normal liver, immortalized by stable transfection with the hTERT [55], and was reported to be liver-specific [56]. L-02 hepatocytes were maintained in 1640 RPMI medium containing 10% fetal bovine serum and a 1% mixture of penicillin and streptomycin in a 5% CO_2_ humidified atmosphere at 37 °C. The medium was changed every two days, and the cells were passaged using trypsin.

### 4.3. Treatment of Cells with Cr(VI) and CoQ10

Cells were treated with a final concentration of 1–4 μM K_2_Cr_2_Q_7_ for 24 h in a complete medium. Cells were pretreated for 2 h prior to K_2_Cr_2_Q_7_ exposure and were co-treated for the 24-h K_2_Cr_2_Q_7_ exposure period at a final concentration of 2.5 μM CoQ10. Control samples were exposed to an equivalent concentration of DMSO as the solvent control.

### 4.4. Cell Viability Assay

MTT was used to evaluate cell viability. Cells were seeded in 96-well plates (1 × 10^4^/well) and cultured in the presence of 0, 0.5, 1, 2, 4, or 8 μM Cr(VI) or 0, 1.25, 2.5, 5, 10, or 20 μM CoQ10 for 24 h. After 24 h of incubation, 10 μL of MTT solution (stock solution of 5 mg/mL in PBS) was added to each well of the 96-well plates, and the plates were incubated for an additional 4 h at 37 °C. The MTT-reducing activity of the cells was measured by treatment with DMSO prior to reading at 490 nm with an automatic microplate reader.

### 4.5. Preparation of Mitochondria

The treated cells were washed with PBS and then centrifuged at 600 g for 5 min. The supernatant was discarded, and the pellets were suspended in ice-cold lysis buffer (250 mM sucrose, 20 mM *N*-(2-hydroxyethy)piperazine-*N*′-(2-ethanesulfonic acid (HEPES), pH 7.4, 10 mM KCl, 1.5 mM MgCl_2_, 1 mM each of EGTA, EDTA, DTT, and PMSF, and 10 μg/mL each of leupeptin, aprotinin, and pepstatin A) and incubated for 20 min. The cells were homogenized up and down 20 times, on ice, at 1000 r.p.m., transferred to a new tube, and centrifuged at 600× *g* for 10 min at 4 °C The supernatant was then centrifuged at 12,000× *g* for 10 min at 4 °C. Afterwards, the supernatants were collected and centrifuged at 12,000× *g* for 15 min at 4 °C for preparation of the cytosolic fraction. The precipitated pellets were resuspended in the lysis buffer and were used as the mitochondrial fraction after centrifugation at 12,000× *g* for 10 min [57].

### 4.6. Extraction and Quantification of CoQ10

Extraction was performed as described previously. Briefly, 250 μL of samples and 750 μL of hexane:ethanol (5:2, *v*/*v*) were mixed together for 1 min using a vortex mixer. The mixture was centrifuged for 3 min at 4000× *g*, and 450 μL of the hexane layer was collected, dried under a stream of nitrogen, and dissolved in 100 μL of ethanol (1:1, *v*/*v*) [58]. Quantification of CoQ10 was performed by HPLC according to Lass and Sohal [59]. A 10-μL aliquot of the extract was chromatographed on a reverse-phase C180 HPLC column (150 mm × 4.6 mm, 5 μM; Thermo Hypersil), using a mobile phase consisting of ethanol: methanol (1:1, *v*/*v*) at a flow rate of 0.8 mL/min. The eluent was monitored with a UV detector at 275 nm.

### 4.7. Gene Chip ANALYSIS

Total RNA was isolated using Trigo (Sigma), and first-strand cDNA was synthesized using RevertAid M-MuL V Reverse Transcriptase (Thermo, Waltham, MA, USA). A Genechip 30 IVT Express Kit was used to synthesize double-stranded cDNA for in vitro transcription (IVT, standard Affymetrix procedure, Santa Clara, CA, USA). During the synthesis of the amplified RNA (aRNA), a biotinylated nucleotide analog was incorporated as a label for the message, and the aRNA was purified with magnetic beads. A 15-μg quantity of aRNA was fragmented with a fragmentation buffer according to the manufacturer’s instructions. Next, 15 μg of fragmented aRNA were hybridized with Affymetrix Human Genome U133 plus 2.0 arrays, according to the manufacturer’s instructions. The chips were heated in a GeneChip Hybridization Oven-645 for 16 h at 60 rpm and 45 °C. The chips were washed and stained using a Genechip Fluidics Station-450 with the Affymetrix HWS kit. Chip scanning was performed with an Affymetrix Gene-Chip Scanner-3000-7G, and the normalized data were extracted using Affymetrix GCOS software (1.0, Santa Clara, CA, USA). The normalized spot intensities were transformed to gene expression log 2 ratios, and comparisons between control and treated groups were conducted using a *t*-test. A *p*-value ≤ 0.05 and a fold change value ≥2 indicated statistically significant regulation. A two-fold change was indicated at log2(Ratio) ≥ 1.0 or log2(Ratio) ≤ −1.

### 4.8. Real-Time PCR

Total RNA was isolated using Trigo (Sigma), and the first-strand cDNA was synthesized using RevertAid M-MuL V Reverse Transcriptase (Thermo). The cDNA was amplified in 20-μL reactions using SYBR premix Dye I (TAKARA BIO, Shiga, Japan) in a Thermal Cycler Dice Real-Time System (TAKARA BIO). The mRNA expression was normalized to the expression of the standard reference gene GAPDH. The primer sequences are shown in the Table 2.

### 4.9. Determination of Reactive Oxygen Species (ROS)

Cells were washed three times with a serum-free medium, and thereafter CellROX^®^ Green Reagent (Thermo Fisher, Waltham, MA, USA) was added to the cells at 5 μM final concentration, followed by incubation for 30 min at 37 °C. The cells were then washed twice with PBS and harvested. One hundred microliters of resuspended sample were added to 96-well plates, and ROS levels were assessed by fluorescence spectrophotometry with excitation at 485 nm and emission at 520 nm. Cells on the coverslip were observed under a confocal microscope.

### 4.10. Measurement of Superoxide Anion (O_2_^−^)

The treated cells were incubated with the cellular O_2_^−^-sensitive fluorescent indicator dihydroethidium (DHE) at a final concentration of 5 μM for 30 min at 37 °C, and protected from light. They were washed three times with PBS, collected, resuspended in 2 mL of PBS, and finally examined by fluorescence spectrophotometry with excitation at 535 nm and emission at 610 nm.

### 4.11. Evaluation of Methane Dicarboxylic Aldehyde (MDA) and Superoxide Dismutase (SOD) Levels

MDA levels were determined using the trace MDA detection kit and a microplate reader at 530 nm. SOD levels were measured using the total superoxide dismutase activity assay, which involves the inhibition of superoxide-induced chromogen chemiluminescence by SOD, according to the manufacturer’s instructions. The absorbance of the wells was read using a microplate reader at a primary wavelength of 550 nm.

### 4.12. Measurement of the Mitochondrial Mass

The mitochondrial mass was evaluated using the fluorescent probe 10-*N*-nonyl acridine orange (NAO) as previously described [30]. Treated cells were incubated in a medium containing 5 μM NAO for 30 min at 37 °C and protected from light. The NAO fluorescence intensity was determined using a microplate reader (Gemini EM, Molecular Devices, Sunnyvale, CA, USA). The emission and excitation wavelengths were 530 and 485 nm, respectively.

### 4.13. Measurement of the Mitochondrial Transmembrane Potential (MMP, Δψm) in Cells

The cells were loaded with JC-1 for 20 min at 37 °C. Depolarization of Δψm was assessed by measuring the fluorescence intensities at excitation and emission wavelengths of 490 and 539 nm, respectively, to measure JC-1 monomers. An excitation wavelength of 525 nm and an emission wavelength of 590 nm were used to measure JC-1 aggregates using a fluorescence microplate reader. During the measurements, the cells were maintained at 4 °C and protected from light. All fluorescence measurements were corrected by autofluorescence, which was determined using cells not loaded with JC-1.

### 4.14. Measurement of the MPTP Opening Degree

The cells were washed three times with PBS, and calcein-AM was added, followed by incubation for 20 min at 37 °C. Next, the cells were washed twice with GENMED cleaning liquid, harvested, resuspended in a cleaning liquid, and finally examined by fluorescence spectrophotometry with excitation at 488 nm and emission at 505 nm.

### 4.15. Measurement of Intracellular ATP Levels

The level of ATP was examined using the ATP assay kit (S0026, Beyotime, Shanghai, China), which utilized the catalysis of firefly luciferase to generate fluorescence requiring ATP to develop an ATP quantified method. The measurement was performed following the manufacturer’s instructions. The absorbance was detected using a Luminometer.

### 4.16. Measurement of the Cellular Calcium Concentration (Ca^2+^)

Cultured cells were incubated with the cellular Ca^2+^-sensitive fluorescence indicator Fluo-3AM at a final concentration of 2.5 μM for 30 min at 37 °C and protected from light. The cells were subsequently washed three times with PBS, collected, and resuspended in 2 mL of PBS for posterior examination by fluorescence spectrophotometry with excitation at 488 nm and emission at 525 nm.

### 4.17. Caspase Activity Assay

Caspase levels were measured with caspase-3 and caspase-9 activity assay kits according to the manufacturer’s instructions (Beyotime). The absorbance was measured on a microplate reader at 405 nm, and the caspase activities were subsequently calculated based on the absorbance.

### 4.18. Western Blot Analysis

For Western blot analysis, cytosolic and mitochondrial fractions were prepared as reported previously. Samples containing an equal amount of concentrated proteins were separated on 10% gradient SDS-polyacrylamide gel and transferred to a polyvinylidene difluoride membrane by electroblotting for 90 min at 100 V and 4 °C. Non-specific membrane binding sites were blocked with blocking solution (PBS, 0.5% Tween-20, pH 7.4), containing 5% non-fat dry milk for 1 h at 4 °C. The membrane was incubated with primary mouse anti-Cyt c monoclonal antibody (Abcam, Cambridge, UK) diluted 1:1500 or goat anti-rabbit Bcl-2 (1:500, Santa Cruz, Santa Cruz, CA, USA) and Bax antibody (1:500, Santa Cruz) in blocking solution overnight at 4 °C. The membrane was washed thoroughly with PBS-T and then incubated for 1 h with horseradish peroxidase-conjugated anti-mouse (1:4000; Santa Cruz) or anti-rabbit IgG antibody (1:6000; Santa Cruz) in blocking solution, detected by chemiluminescence reagent plus, and exposed to film.

### 4.19. Immunofluorescence

Cyt c translocation from mitochondria to cytoplasm was analyzed by immunofluorescence. Treated cells were incubated with Mito-Tracker Red (Thermo Fisher) at 500 nM for 45 min, washed twice with PBS, and with 2% paraformaldehyde for 20 min at room temperature. The cells were blocked with blocking solution (1% BSA, 0.15% saponin and 10% goat serum in PBS) for 30 min at room temperature, incubated with primary antibody specific for Cyt c (1:200) overnight at 4 °C, and then incubated with secondary antibody for 1 h at room temperature. The cells were washed three times with PBS, and then the nuclei were stained with Hoechst for 5 min. Images were captured with a confocal microscope.

### 4.20. FITC Annexin V/propidium Iodide (PI) Staining for Apoptotic Cells

Cells were washed three times with PBS before suspension in binding buffer, and 10 μL of Annexin V-FITC were mixed with 200 μL of cell suspension containing 10^6^ cells. The cells were incubated at room temperature for 30 min and shielded from light. Then, 10 μL of PI solution were added to the cells and they were incubated for 10 min on ice. The scatter parameters of the cells were analyzed using a flow cytometer. Usually, four cell populations are identified by the flow cytometer. The viable population is displayed in the lower-left quadrant; the early apoptotic population and late apoptotic population are presented in the lower-right quadrant and in the upper-right quadrant, respectively. Signals in the upper-left quadrant present a necrotic population.

### 4.21. Protein Assay

All protein assays in this study were measured using a Q5000 UV-Vis spectrophotometer (Quawell, Sunnyvale, CA, USA).

### 4.22. Statistical Analysis

All data were expressed as the group mean ± SD. Comparisons between control and treated groups were conducted using one-way ANOVA, as appropriate, followed by LSD. A value of *p* < 0.05 was considered to indicate statistical significance. Statistical analysis of the data was performed using SPSS18.0. All experiments were performed three times.

## 5. Conclusions

In conclusion, we have demonstrated that Cr(VI) may induce mitochondrial CoQ10 deficiency by inhibiting the expression of genes involved in the CoQ10 biosynthesis pathway, and by subsequently causing oxidative stress, altering the mitochondrial network, and reducing mitochondrial biogenesis. CoQ10 exerts a potent protective effect against Cr(VI)-induced apoptosis, reduces intracellular oxidative stress, inhibits mitochondrial depolarization, increases mitochondrial mass and the mtDNA copy number, decreases the degree of MPTP opening, ameliorates caspase activity, equilibrates Bcl-2/Bax expression, and antagonizes subsequent apoptosis. We conclude that Cr(VI)-induced CoQ10 deficiency might be corrected by supplementation with CoQ10, which appears to stimulate mitochondrial biogenesis and prevent apoptosis. Thus, new perspectives on mitochondrial toxicity related to Cr(VI) exposure are suggested, and we provide new insights into therapeutic potentials and strategies for protecting hepatocytes against Cr(VI)-induced oxidative stress and apoptosis.

## Figures and Tables

**Table 1:** Effect of Cr(VI) on the expression of genes involved in the CoQ10 biosynthesis pathway in L-02 hepatocytes.

Gene Name	Description	log2(Ratio)	*p*-Values
*PDSS1*	prenyl (decaprenyl) diphosphate synthase, subunit 1	0.079217	0.728622
*PDSS2*	prenyl (decaprenyl) diphosphate synthase, subunit 2	−0.917213	2.86 × 10^−5^
*COQ2*	coenzyme Q2 homolog, prenyltransferase	−0.136864	0.434046
*COQ3*	coenzyme Q3 homolog, methyltransferase	−0.443767	0.002707
*COQ4*	coenzyme Q4 homolog	−0.646236	0.004524
*COQ5*	coenzyme Q5 homolog, methyltransferase	−0.986167	4.4 × 10^−8^
*COQ6*	coenzyme Q6 homolog, monooxygenase	−0.185303	0.267227
*COQ7*	coenzyme Q7 homolog, ubiquinone	−0.308896	0.011926
*COQ7*	coenzyme Q7 homolog, ubiquinone	−0.528129	0.115161
*ADCK3*	aarF domain containing kinase 3	0.970573	6.12 × 10^−9^
*ADCK4*	aarF domain containing kinase 4	NA	NA
*COQ9*	coenzyme Q9 homolog	−0.936477	3.1 × 10^−18^
*COQ10A*	coenzyme Q10 homolog A	0.239029	0.045092
*COQ10B*	coenzyme Q10 homolog B	0.59875	0.000958
*APTX*	aprataxin	−0.648958	0.003698
*ETFDH*	electron-transferring-flavoprotein dehydrogenase	−1.412843	4.43 × 10^−7^
*BRAF*	B-Raf proto-oncogene, serine/threonine kinase	−0.837738	0.008798
*PMVK*	phosphomevalonate kinase	−0.146285	0.455768
*MVD*	mevalonate (diphospho) decarboxylase	0.842443	0.01009
*MVK*	mevalonate kinase	0.612375	0.000892

Two-fold change indicates log2(Ratio) ≥1.0 or log2(Ratio) ≤−1; log2(Ratio) = “NA” indicates that the difference in intensity between the two samples was ≥1000.0, *n* = 3.

**Table 2:** Primer sequences.

Target	Forward Primer (5′→3′)	Reverse Primer (5′→3′)
mtDNA	CAAACCTACGCCAAAATCCA	GAAATGAATGAGCCTACAGA
GAPDH	TGACAACAGCCTCAAGAT	GAGTCCTTCCACGATACC

## Abbreviations

Cr(VI)	hexavalent chromium
CoQ10	coenzyme Q10
ROS	reactive oxygen species
MMP	mitochondrial membrane potential
Cyt c	cytochrome c
MPTP	mitochondrial permeability transition pore
MDA	methane dicarboxylic aldehyde
SOD	superoxide dismutase
PDSS1	prenyl(decaprenyl) diphosphate synthase, subunit 1
PDSS2	prenyl (decaprenyl) diphosphate synthase, subunit 2
COQ2	4-hydroxybenzoate polyprenyltransferase
COQ3	coenzyme Q3 methyltransferase
COQ4	coenzyme Q4
COQ5	coenzyme Q5 methyltransferase
COQ6	coenzyme Q6 monooxygenase
COQ7	coenzyme Q7 homolog
ADCK3	aarF domain-containing kinase 3
ADCK4	aarF domain-containing kinase 4
COQ9	coenzyme Q9
COQ10A	coenzyme Q10A
COA10B	coenzyme Q10B
APTX	aprataxin
ETFDH	electron transfer flavoprotein dehydrogenase
BRAF	B-Raf proto-oncogene, serine/threonine kinase
PMVK	phosphomevalonate kinase
MVD	mevalonate diphosphate decarboxylase
MVK	mevalonate kinase
